# Are Daycare Workers at a Higher Risk of Parvovirus B19 Infection? A Systematic Review and Meta-Analysis

**DOI:** 10.3390/ijerph16081392

**Published:** 2019-04-17

**Authors:** Karla Romero Starke, Marlen Kofahl, Alice Freiberg, Melanie Schubert, Mascha Luisa Groß, Stefanie Schmauder, Janice Hegewald, Daniel Kämpf, Johanna Stranzinger, Albert Nienhaus, Andreas Seidler

**Affiliations:** 1 Institute and Policlinic of Occupational and Social Medicine (IPAS), Faculty of Medicine Carl Gustav Carus, Technische Universität Dresden, 01307 Dresden, Germany; marlen.kofahl@tu-dresden.de (M.K.); alice.freiberg@tu-dresden.de (A.F.); melanie.schubert@tu-dresden.de (M.S.); mascha1806@gmail.com (M.L.G.); stefanie.schmauder@tu-dresden.de (S.S.); janice.hegewald@tu-dresden.de (J.H.); Daniel.Kaempf@mailbox.tu-dresden.de (D.K.); andreas.seidler@mailbox.tu-dresden.de (A.S.); 2 Section Occupational Health, Basic Principles of Prevention and Rehabilitation, Institution for Statutory Social Accident Insurance and Prevention in the Health Care and Welfare Services (BGW), 22089 Hamburg, Germany; Johanna.Stranzinger@bgw-online.de (J.S.); Albert.Nienhaus@bgw-online.de (A.N.); 3 Competence Centre for Epidemiology and Health Services Research for Healthcare Professionals (CVcare), University Medical Centre Hamburg-Eppendorf (UKE), 20251 Hamburg, Germany

**Keywords:** parvovirus B19, fifth disease, daycare workers, daycare, kindergarten teachers, occupational risk, occupational disease

## Abstract

*Objective:* In this systematic review, we aimed to summarize the evidence on the association between being a daycare educator working with children and the possible increased risk of parvovirus B19 infection compared to the general population. *Methods:* The Medline and Embase databases were searched using a defined search to find studies published since 2000. Two reviewers evaluated the search hits using predefined inclusion and exclusion criteria. The resulting studies were extracted and were assessed in eight domains of bias. A pooled relative risk (RR) of parvovirus infection for daycare workers compared to the general population was calculated. *Results:* After evaluating the 7781 search hits and manual search, four methodologically-adequate studies were identified: three cross-sectional studies and one retrospective cohort study. Of the three studies investigating the risk of infection, one evaluated parvovirus B19 seroconversion rates for daycare workers. There was an indication for an increased risk for daycare workers compared to the unexposed population (RR = 1.12, 95% CI 0.98–1.27) using prevalence estimators. Furthermore, daycare workers had a higher seroconversion rate compared to the unexposed population (RR = 2.63, 95% CI 1.27–5.45) in the low risk of bias study. *Conclusions:* Our findings suggest a higher risk of parvovirus B19 infection for daycare workers compared to an unexposed comparison population, which necessitate preventative efforts. Considering the underestimation of the occupational seroconversion risk by prevalence-based estimators, parvovirus B19 infections among daycare workers might mostly be occupationally acquired.

## 1. Introduction

Infection of parvovirus B19 is mostly asymptomatic in children and teenagers, but the “fifth disease” can be the clinical presentation of infection. It is characterized by fever and malaise early in the infection, and by a “slapped-cheek” rash and rheumatic symptoms two weeks after infection, when the appearance of antiviral antibodies occurs [1]. However, infection in women of childbearing age deserves additional attention due to its potentially harmful effects on pregnant women and their fetus. It has been reported that 1–5% of pregnant women are affected by the parvovirus B19 infection [2]. About half of fetal infections are asymptomatic [3], but infection with parvovirus B19 during pregnancy, especially during the first two trimesters, has been associated with a risk of miscarriage, intrauterine fetal death, fetal anemia and non-immune hydrops [4,5,6]. The risk of acquiring parvovirus B19 infection during pregnancy varies between 0.61% in Belgium to 1.58% in Poland [7] and is higher during epidemics.

Parvovirus B19 is globally widespread [4], but nonetheless, the seroprevalence varies by country. The Parvovirus B19 infection is common in childhood, with seroprevalences in Europe increasing quickly through childhood and the teenage years to above 50–70%, followed by a plateau in young adulthood, and another increase starting at 25–30 years old [7,8]. The seroprevalence is also influenced by education, a proxy for socio-economic status [9]. In Germany, Parvovirus B19 seroprevalence in asymptomatic pregnant women varied between 65 and 70%, depending on the year studied (1997–2004) [8]. The epidemic cycles are around 4 years, with 1 or 2 epidemic years, followed by 2 or 3 years of less frequent cases [8].

Transmission through respiratory droplets or by blood [1] usually occurs immediately before the clinical manifestation is present [10]. Contact with small children poses a greater risk for the parvovirus B19 infection [9] since they are more likely to drool. It is, therefore, worthwhile to investigate the risks of women working in close and frequent contact with small children, such as daycare workers or kindergarten teachers. Elsner and colleagues systematically searched and summarized research on various infections in daycare workers in 2009 [11], but to our knowledge, to this date, there has been no systematic review and meta-analysis focusing on the potential risk of parvovirus B19 in daycare workers. The aim of this study was to summarize the evidence of methodologically-adequate studies to investigate if there is an increased risk of parvovirus B19 infection for daycare workers compared to the general population and to quantify this risk. Depending on the size of the increased risk, consideration of the parvovirus B19 infection as an occupational disease in daycare workers could be made. Consequently, we performed a systematic literature review and meta-analysis of studies examining parvovirus B19 infection on daycare workers.

## 2. Methods

### 2.1. Protocol and Registration

Our review on parvovirus B19 infection is part of a comprehensive review of seroprevalences and risks for infections in daycare workers, and an update of a previously-published review on risk of infectious diseases in daycare workers [11]. The guidelines for conducting and reporting meta-analyses of observational studies in epidemiology (MOOSE) [12] and the Preferred Reporting Items for Systematic reviews and Meta-Analyses (PRISMA) [13] were followed. The study protocol was registered in PROSPERO under record number CRD42018083646 and is available at https://www.crd.york.ac.uk/prospero/display_record.php?RecordID=83646.

### 2.2. Search, Selection, and Data Extraction

**Figure 1:**
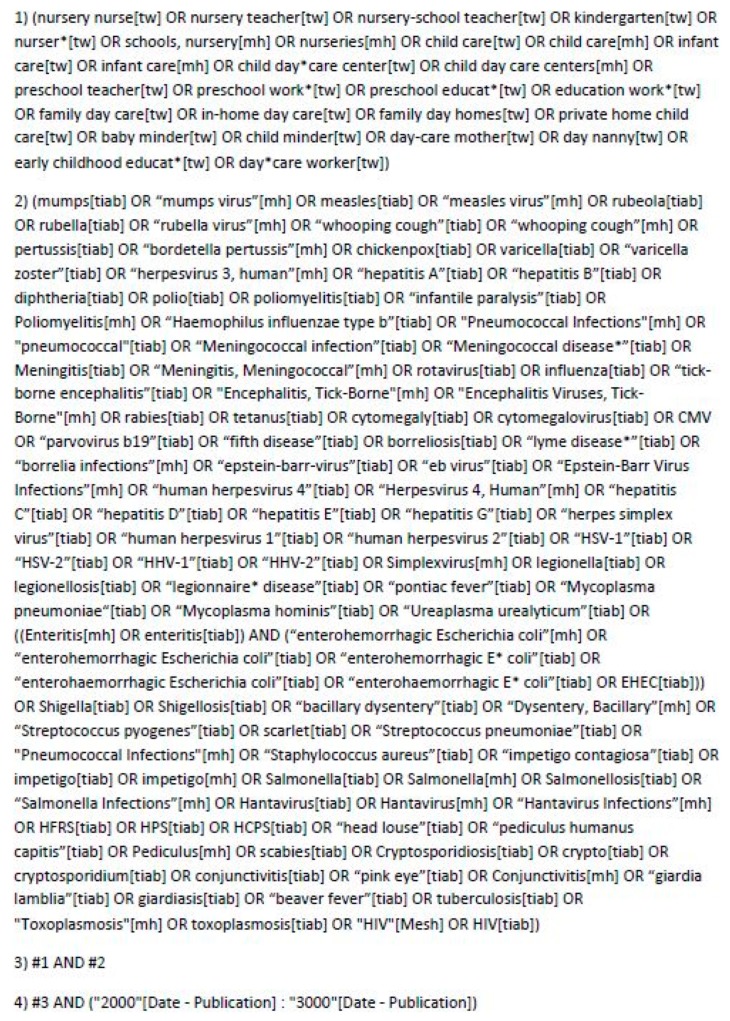
The Medline search strategy PubMed February 2018 (Last update 4 October 2018).

Our aim was to investigate whether daycare workers have an increased risk of a work-related parvovirus B19 infection compared to the general population. On February 14, 2018, we applied a systematic electronic literature search based on the Population, Intervention (Exposure), Control/Comparison, Outcome (PICO) scheme [14] using the Medline and Embase databases to find studies in peer-reviewed journals since 2000, as an update to the previous review [11]. We updated the search on October 4, 2018. The Medline search strategy is a search for several infections, including parvovirus B19, and is shown in Figure 1.

To increase the comparability of the population data, we limited the review to studies in European countries, the USA, Canada, Australia, and New Zealand. We also did not apply language restrictions with the intention to minimize reporting biases. Only cohort, case-control, or cross-sectional studies studying daycare workers between 16 and 70 years old, who worked with children up to 6 years old, were included. For risk of infection, we considered studies using comparison populations employed in other occupational groups where an average population-based risk for infection can be presumed (office workers or the general population), and we excluded studies using occupational groups with an elevated risk of infection as comparison populations (health care workers). We also excluded studies done in response to a disease outbreak in a child daycare center.

Our primary outcome of interest was “parvovirus B19 infection risk” which is best defined as the parvovirus B19 seroconversion rate of daycare workers compared to the unexposed population. Ideally, the daycare workers and comparison group would be tested for parvovirus B19 seropositivity at the beginning of the study. Those who were seronegative would be followed-up for a period of time and the parvovirus B19 seroconversion rates would be compared between both groups after the follow-up period. However, it is likely that many studies may not have investigated seroconversion rates. We, therefore, considered studies in which parvovirus B19 seropositivity is measured for both study groups and a cross-sectional analysis is followed using prevalence ratios or prevalence odds ratios. We also considered studies reporting only parvovirus B19 seroprevalence in daycare workers (lacking a comparison group) as the last option. Together, we considered three outcomes for our study in order of decreasing relevance: parvovirus B19 infection risks measured by seroconversion rate ratios (outcome Ia), parvovirus B19 prevalence ratios or prevalence odds ratios (outcome Ib), and parvovirus B19 seroprevalence of only daycare workers (outcome II).

Two independent scientists screened titles and abstracts of the studies in order to exclude studies unrelated to the *a priori* defined research questions. If there was disagreement on inclusion, a consensus decision was sought by discussion. If still no agreement was achieved, the decision was made by a third reviewer. In the manual search, we screened key articles and systematic reviews to identify further relevant studies. In addition, we used the “citation tracking factor” by Google scholar on key papers to further identify pertinent studies [15,16]. The full texts of the remaining studies were then thoroughly and independently examined by two reviewers to decide whether the inclusion criteria were met. 

The data extraction was done by one reviewer and was checked by a second one. We discussed disagreements in consensus conferences moderated by the principal investigator (K.R.). Whenever there was missing or unclear information, we tried to obtain it through personal communication with authors. The data extraction form includes information on relevant study characteristics (first author and publication year, country of origin, exposure, outcome, study population, recruitment, confounding, analysis method, study results, conflicts of interest, and risk of bias). 

### 2.3. Risk of Bias Assessment

For each included study, two reviewers assessed the risk of bias as high, low, or unclear against eight domains of bias, similar to Ijaz et al. 2013 [17] and Shamliyan et al. 2011 [18]. We also used the criteria described by SIGN (Scottish Intercollegiate Guidelines Network) [19] and CASP (Critical Appraisal Skills Programme of the British National Health Service Appraisal Tools) [20]. The eight domains of bias are presented in the Supplementary Materials.

### 2.4. Overall Assessment of Risk of Bias

For the overall assessment of the risk of bias of a study, we assigned domains into two hierarchical groups. Five domains were defined as “major domains for risk of bias”: (i) recruitment procedure and follow-up (in cohort studies), (ii) exposure definition and measurement, (iii) outcome source and validation, (iv) confounding, and (v) methods of analysis. The other three domains were defined as minor domains and were (vi) chronology (in the case of seroconversion rates and infection risk outcomes), (vii) funding, and (viii) conflicts of interest. Since it is unlikely that parvovirus B19 seropositivity affects whether a person works as a daycare worker, cross-sectional studies should be acceptable in studying risk effects. We, therefore, considered chronology as a minor domain. In order for a study to have an overall low risk of bias, every major domain for risk of bias would have to be rated as low risk. If one of the major domains for risk of bias was rated as either high risk or unclear risk, the study was considered to have a high overall risk of bias. The detailed form is available in Supplementary Table S1.

### 2.5. Statistical Analysis

For the meta-analysis, we analyzed parvovirus B19 prevalence ratios and seroconversion rate ratios to estimate relative risks, as well as seroprevalences. We assessed statistical heterogeneity with the I^2^ statistic, and assessed publication bias by observing the funnel plot asymmetry and performing Egger’s test to check small study bias.

### 2.6. GRADE: Quality of Evidence Assessment

We used the GRADE approach for grading the quality of the total body of evidence [21], following the examples of Ijaz et al. [17] and Kuijer et al. [22]. Four levels of quality were considered: high, moderate, low, and very low. An initial “high” level would indicate having longitudinal studies assessing seroconversion rate ratios. If only cross-sectional analyses were done, then the starting level would be set to “moderate”. The quality of evidence was downgraded based on these 4 factors: study limitations (the majority of studies having a high risk of bias), inconsistency (I^2^ > 50%), imprecision (range of the CI of studies > 2.0), and publication bias (yes or unclear). Study findings with large effect sizes (an effect estimate >1.5 in low risk of bias studies) resulted in an upgrade of the quality of evidence.

## 3. Results

### 3.1. Search Results

**Figure 2:**
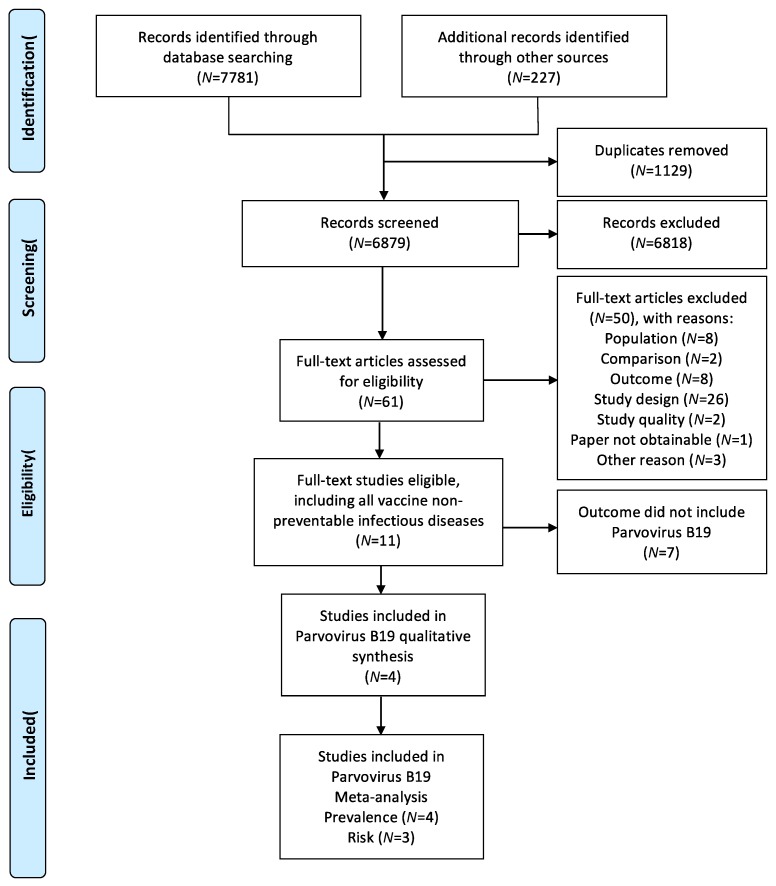
The study process flow.

The database search identified 7781 studies, and the manual search identified an additional 227 studies. After the removal of duplicates, 6879 studies were screened and 61 studies were assessed for eligibility. Of the 11 eligible studies, four studies [23,24,25,26] investigated parvovirus B19. The process flow is shown in Figure 2, and the four studies are characterized in Table 1 and below. The studies originated either in Europe [23,24,25] or in Canada [26]. One study investigated seroconversion rate ratios [24] (Outcome Ia) and had an overall low risk of bias, while two studies with a high risk of bias [23,25] reported prevalence ratios (Outcome Ib). All four studies reported parvovirus B19 seroprevalence.

The cross-sectional study of De Villemeur et al. [23] recruited 395 women working in 1 of 83 child daycare centers and 382 women employed in 1 of 2 business organizations in Isère, France, for the comparison group. The response for the daycare workers was acceptable (63%). Although no specific information was available regarding the response of the business workers, they were required to have annual health and blood tests, where they were recruited. No refusals were reported for them (email communication with the author). This study included personnel in the exposed population who were not in regular contact with children (cleaning or administrative personnel). Parvovirus B19 seroprevalence was measured by B19 Ig G assays for both groups. The resulting seroprevalence of parvovirus B19 in childcare workers was 79.4% (Table 2), while the comparison had a prevalence of 68.0%. Stratification by age resulted in a lower prevalence for women who were 37 years old or younger (67.7%, 95% CI 59.0–75.5) than women who were older than 37 years of age (85.4%, 95% CI 80.6–89.5). Using multivariate log-binomial models (adjusted by age, occupational group, number of children, duration of in-home childcare, informal child-care, number of years in a child-care facility, and residency in a low developed country) there was a slightly increased risk of infection for daycare workers compared to the business workers (Prevalence Ratio, PR = 1.05, 95% CI 0.94–1.16), although it was not statistically significant.

Using retrospective data collected between 1992 and 1993 from the Finnish Maternity Cohort, Riipinen et al. [24] investigated parvovirus B19 infection risk on pregnant daycare workers. Although this study coincided with a major epidemic of parvovirus B19, it was not conducted in response to a disease outbreak in a child daycare center, and thus it was included. Seroprevalence was measured using early-pregnancy sera, and the cord blood.

Samples of seronegative women were tested for seroconversion during pregnancy. Although the authors used healthcare workers as the reference group to estimate the increased risk of daycare workers, they also included another comparison group “other occupations”, which can be used to indirectly compare the daycare workers to the general population. The inclusion of both comparison groups thus qualified Riipinen et al. for inclusion. For the reporting of seroprevalence, education (daycare education or healthcare education) was used as a proxy for occupation. For seroconversions, occupations were identified from the files of professional unions and verified through the register of the Finnish Center for Pensions, and the duration of employment was provided. The study is characterized by a large sample size with a high response and an acceptable loss to follow-up. Pregnant daycare workers had a 59.1% B19 parvovirus seroprevalence, while 54.9% of pregnant healthcare workers were seropositive. There was no information given on the seroprevalence of pregnant workers in other occupations. Seroprevalence increased slightly with age. The daycare workers had an annual seroconversion rate of 12.2%, while those in healthcare had annual seroconversion rates of 6.7%. Compared to healthcare workers, daycare workers had a higher rate for infection (HR = 2.63, 95% CI 1.27–5.46), and workers in other professions were at a decreased risk of infection compared to healthcare workers (HR = 0.85, 95% CI 0.18–4.09). When the analysis was restricted to nulliparous women with no childcare leave, the risk of infection for daycare workers compared to healthcare workers increased (HR = 5.59, 95% CI 1.40–22.4).

In a cross-sectional study, van Rijckevorsel et al. 2012 [25] studied the parvovirus B19 seroprevalence among 242 females working in one of 38 randomly-sampled daycare centers in 2007 in Amsterdam, measured by parvovirus B19 IgG ELISA. Their risk of infection was compared to women who participated in a cross-sectional survey of the Amsterdam population (Amsterdam Health Monitor, AHM) in 2004. The response of daycare centers was appropriate (60%), and it was reported that nearly all daycare workers had agreed to participate. The AHM response (including available blood samples) was low (43.8%) and was lowest for the youngest age group (18–34 years) [27,28]. Parvovirus B19 seroprevalence for daycare workers was 72.7%, while the comparison group had a 60.4% seroprevalence. A multivariate binomial regression analysis adjusting for age, country of birth, and having children resulted in an increased prevalence ratio for daycare workers compared to the AHM population (PR = 1.2, 95% CI 1.1–1.4). No adjustment was done for socioeconomic status.

Educators working in 2001 in Canadian daycare centers which enrolled children under 36 months were studied by Gilbert et al. [26]. To be eligible, the educators must have worked for at least 15 h a week caring for children less than 60 months of age. The response was low, and a substantial differential selection of the study population could not be excluded. Through questionnaires, characteristics of the daycare workers, including their duration of employment, were obtained. The overall seroprevalence for daycare workers was 69.8% (95% CI 65.5–73.9), and seroprevalence appeared to increase with age and with increasing experience in daycare.

**Figure 3:**
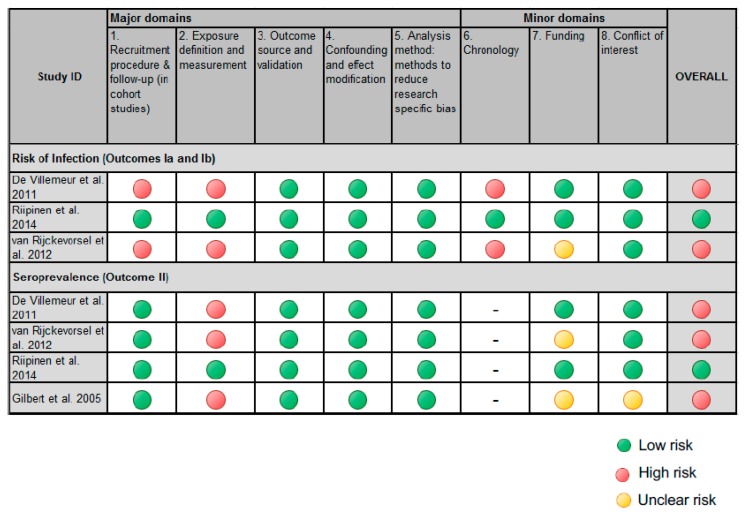
The risk of bias of the included studies.

A summary of the studies’ risk of bias is presented in Figure 3, and a synopsis of their effect estimates is shown in Table 2.

### 3.2. Risk of Infection (Outcomes Ia and Ib)

**Figure 4:**
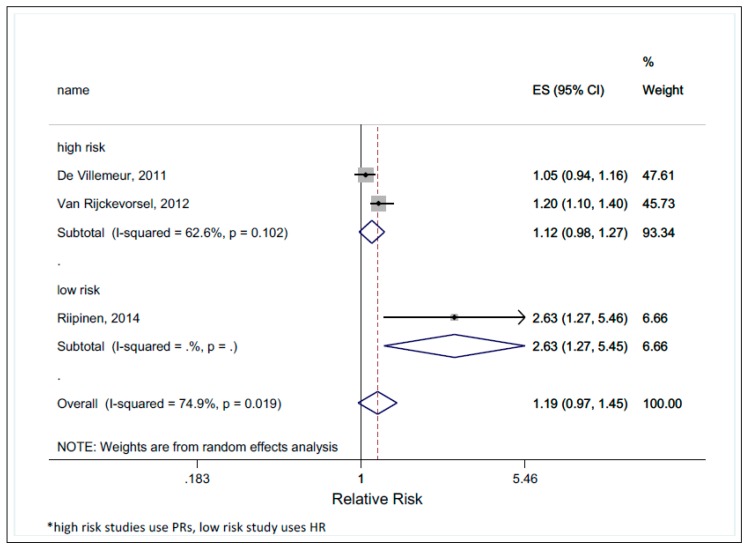
The effect size (ES) of relative risk by study’s risk of bias.

Only one study [24] providing the seroconversion risk ratio (outcome Ia) of daycare workers compared to another population was identified. Two other studies [23,25] presented prevalence ratios. We compared the studies presenting PRs (also with high risks of bias) with the study providing a seroconversion risk ratio (low risk of bias). The pooled relative risk for the studies reporting PRs was 1.12 (95% CI 0.98–1.27), much lower than the one reporting the seroconversion risk ratio RR 2.63 (95% CI 1.27–5.45) (Figure 4).

The funnel plot did not show evidence of publication bias (Figure S1 in Supplementary Materials), and the Egger’s test was not significant (Egger’s coefficient = 2.92, 95% CI −17.5–23.4, *p* = 0.32).

### 3.3. Parvovirus B19 Seroprevalence (Outcome II)

All four studies were included in the meta-analysis for seroprevalence. The overall prevalence of parvovirus B19 in daycare workers for the four included studies was 70.2% (95% CI 59.9–80.4) and heterogeneous (I^2^ = 96.5%). To study whether the variation between studies might be due to age differences in the study populations, we analyzed older and younger populations separately. Van Rijckevorsel [25] did not categorize by age, and they were excluded from this analysis. De Villemeur [23] stratified their results by two age categories (≤37 vs >37 years), and we categorized each as “younger” or “older”, respectively. The results of the rest of the studies could be categorized by age in a similar way (≤34 years “younger” vs. >34 years “older”). The pooled prevalence for the younger population was 62.7% (95% CI 57.2–68.3, I^2^ = 68.5%), and that for the older population was 74.4% (95% CI 61.2–87.5, I^2^ = 93.6%). The pooled seroprevalence for the high risk of bias studies [23,25,26] was 74.1% (95% CI 67.9–80.3, I^2^ = 81.2%), while the low risk of bias study [24] had a seroprevalence of 59.1% (95% CI 56.9–61.3). The forest plots for seroprevalence are available in the Supplementary Materials (Figures S2 and S3).

After starting our GRADE evaluation at “high”, we judged that the quality of evidence for risk of infection of daycare workers to be “moderate”. The majority of studies had a high risk of bias and there was a high inconsistency between the studies. However, the imprecision in the studies was acceptable, and the effect estimate in the low risk of bias study was greater than 1.5.

## 4. Discussion

To our knowledge, this is the first comprehensive systematic review on the parvovirus B19 infection on daycare workers since 2009. Altogether, we found moderate evidence of an increased parvovirus B19 seroprevalence for daycare workers compared to the unexposed population.

We also found the parvovirus B19 seroprevalence for daycare workers to be 70%.

Analysis of seroprevalence by age revealed a lower seroprevalence for younger workers compared to their older counterparts. In Germany, the parvovirus B19 seroprevalence has been reported to be at 20% in 1 to 3-year-old children, and at 67% in adolescents, rising to 72% in adulthood to 79.1% in the elderly [29].

### 4.1. Agreement with Other Studies and Reviews

The previous review by Elsner et al. [11] also concluded that daycare workers were at a higher risk for parvovirus B19 infection even if our inclusion criteria differed significantly, in particular, with respect to our more stringent criteria regarding the exposure definition, exclusion of studies done as a result of an outbreak, and recruitment procedure. Because Elsner et al. investigated studies published before 2009, and due to our differing criteria, there was no overlap in studies between both reviews other than a cross-sectional study on parvovirus B19 seroprevalence [26].

### 4.2. Strengths and Limitations

The main strengths of our research methods were the systematic literature search using a comprehensive search string in two databases (Medline and Embase), the independent appraisal of titles, abstracts, and full texts by two researchers, and the dual assessment of study quality with consensus finding to determine the final quality score in case of disagreements. We performed a formal risk of bias assessment for the studies and integrated these assessments for our analysis and conclusions. Because no reliable information about the infection risk or prevalence of a definable population can be determined, studies using a convenience sample were excluded. Similarly, studies with no information on the response (%) were excluded due to the potential for selection bias. Furthermore, only peer-reviewed articles published in scientific journals were included. The resulting exclusion of the grey literature could mean a loss of information. However, including studies with inadequate quality would have introduced bias.

We excluded studies that were unclear as to whether the person was working in a daycare center setting or as a childminder with children up to six years old. Such studies may have introduced other exposed populations, such as healthcare workers working with children.

### 4.3. The Methodological Quality of Included Studies

Only one of the four included studies were assessed as having a low risk of bias. Studies were rated with a high risk of bias mainly due to two domains: recruitment procedure and exposure definition. Several studies either had low responses in either the exposed or unexposed populations, or the information was not provided. In addition, there were studies in which the exposed population comprised of both daycare workers in contact with children and other daycare workers with no contact with children (such as administrators or cleaning staff). This misclassification of the exposure could have resulted in an underestimation of the risk for daycare workers. In other studies, no information on the job duties or duration of employment was collected, both of which could have explained differing seroprevalence within and between the populations. Furthermore, one of the studies [25] investigating relative risks of infection did not adjust for socioeconomic status. Although the unexposed population used in this study was based on the general population, there still may have been socioeconomic differences between the daycare workers and the unexposed population. Two studies investigated the risk of infection of daycare workers compared to an unexposed population using a cross-sectional analysis. In these studies, no difference between parvovirus B19 immunity and a fresh infection could be made because IgM was not measured.

There is merit in a further discussion of Riipinen et al. 2014 as the authors used seroconversion rate ratios to compare risks between daycare workers and two other populations: healthcare workers (the reference group) and “other occupations”. Daycare workers had more than twice the seroconversion rate than healthcare workers, while those in “other occupations” had a 15% lower rate than healthcare workers. Because healthcare workers are already more at risk than the general population (“other occupations”), the reported risk (RR = 2.63) is underestimated and the actual risk of daycare workers compared to the general population will be higher.

We should also address the pooled size of effect estimated by prevalence ratios in two out of three studies [23,25]. It is important to note that parvovirus B19 seroprevalence in the late teen or young adulthood years, at the start of the women’s working life, is already high. The seroconversion risk will likely be underestimated when using prevalence ratios because the “non-occupational” seroprevalence cannot be removed from the prevalence-based “occupational” risk estimates. As an example, if an unexposed group had a parvovirus B19 seroprevalence of 65%, then the daycare workers would have a seroprevalence of 72.8%, assuming a PR of 1.12 (pooled from both studies reporting PRs as seen in Figure 3). If we would subtract 60% (a reasonable estimate of seropositivity at the start of the working life [7,8,29]) from both groups, we would obtain a seroconversion of 5% for the unexposed group and 12.8% for the daycare workers, which would, in turn, result in a risk effect greater than 2.5. In order to be considered an occupational disease, the risk of infection for the exposed group should be at least double than that of the unexposed group, corresponding to at least a 50% probability of causation. Taking the above examples, our results show the potential of parvovirus B19 infection as an occupational disease for daycare workers.

A German study [30] estimated that 1.4 deaths and 1.7 cases of hydrops fetalis would be prevented by an employment ban of seronegative pregnant women working in daycares with children below six years of age until the 34th week of pregnancy. An aggressive hygiene campaign stressing frequent handwashing and cleaning of contaminated surfaces could also reduce the risk of parvovirus B19 infection.

### 4.4. Implications for Practice and Research

This review points to a higher risk of parvovirus B19 infection workers compared to a comparison population. After adjustment for confounders such as age, socio-economic status (SES), and having one’s own children, the low-risk of bias study showed a higher risk of seroconversion for daycare workers compared to a comparison population. Cross-sectional studies also point to a higher risk of infection. Since there is no vaccine available, informing daycare workers of their immunity status, raising awareness of the potential risk of infection in women trying to conceive or during pregnancy, and reinforcing prevention measures such as washing hands regularly and cleaning of contaminated surfaces may decrease the risk of infection. In case of a confirmed pregnancy in a seronegative daycare worker, necessary precautions should be taken to prevent infection. Considering the underestimation of the occupational seroconversion risk by prevalence-based estimators, parvovirus B19 infections among daycare workers might mostly be occupationally acquired.

## 5. Conclusions

Our findings suggest a higher risk of parvovirus B19 infection for daycare workers compared to an unexposed comparison population, which necessitate preventative efforts. Considering the underestimation of the occupational seroconversion risk by prevalence-based estimators, parvovirus B19 infections among daycare workers might mostly be occupationally acquired.

## Figures and Tables

**Table 1:** The characteristics of the included studies.

First Author, Publication Year	Study Region	Study Design	Population			Exposure, Duration of Employment, Job Duties	Outcome Measurement
			Sample Population	No. of Exposed/ No. of Unexposed (Response, Age) Follow-up (%)	Time of Recruitment		
De Villemeur et al. 2011	France	Cross-sectional study	Women 20–50 yr, not pregnant, employed in either 1 of 83 child daycare centers (exposed) or in 1 of 2 business organizations (unexposed)	Exposed *N* = 395 (all women) Age characteristics: Mean: 39.0 yr Range: 38.3–39.7 yr Response: 63.3% Not exposed *N* = 382 (all women) Age characteristics: Mean: 39.3 yr Range: 33.1–34.8 yr Response: N.A. ^†^	2005–2007	Self-administered questionnaires Duration of employment: N.A. Job duties: N.A.	Blood samples EIA^®^ anti-B19 IgG (Biotrin)
Riipinen et al. 2014	Finland	Retrospective cohort study	Pregnant women from the Finnish Maternity Cohort, who were pregnant during September 1992–August 1993	**Serological analysis (baseline)** Exposed *N* = 1162 (all women) Age characteristics: Distribution: 19–24 yr: 212 (10.8%) 25–29 yr: 935 (47.6%) 30–34 yr: 638 (32.5%) 35–48 yr: 181 (9.2%) Not exposed *N* = 60 (all women) Age characteristics: not specified, but in reproductive age Response: 96.9% (all groups) **Cord blood samples** **(follow-up)** Exposed *N* = 331 Not exposed *N* = 60 (other occupation) *N* = 326 (healthcare workers) Follow-up: 56.6% (all exposure groups)	September 1992–August 1993	Nursery school teachers identified from files of Trade Union of Education (95% unionization rate for teachers) and the Union of Professional Social Workers. Other occupations identified from the register of National Supervisory Authority for Welfare and Health To define employment-verified occupation, the registers of the Finnish Center for Pensions were used.	B19V IgG by an indirect enzyme immunoassay using streptavidin-bound biotinylated virus-like particles of virus protein 2 as an antigen.
Van Rijckevorsel et al. 2012	The Netherlands	Cross-sectional study	Childcare personnel working in 38 daycare centers on the Amsterdam municipal register (exposed), compared to women not working in daycare from Amsterdam Health Monitor (AMH) survey (unexposed)	Exposed *N* = 242 (all women) Age characteristics: Mean: 29.0 yr Inter-quartile range (IQR): 24–35 yr Response: “nearly all agreed to participate” (~100%) Not exposed *N* = 298 (all women) Age characteristics: Mean: 35 yr IQR: 28–40 yr Response = 43.8%	Daycare center employees: 2007 Unexposed group: 2004	Face interviews and through a cross-sectional survey in 2007 by the Public Health Service of Amsterdam Duration of employment: N.A. Job duties: N.A.	Blood samples NovaLisa Parvovirus B19 recombinant IgG ELISA ^‡^
Gilbert et al. 2005	Canada	Cross-sectional study	Educators working in daycare centers which were in current operation, enrolled children under 36 months and employed at least six educators. Educators had to be employed for at least 15 h/week and cared for children <60 months of age.	Exposed *N* = 477 (all women) Age characteristics:Mean: 34 years Range: 17–70 years <50 yr (91%) Response at daycare level: 53.3% Individual response: 44.3%	October–December 2001	Questionnaires sent to directors and educators Duration of employment <5 yr: *n* = 162 (34.0%) 5–9 yr: *n* = 132 (27.7%) 10–14 yr: *n* = 106 (22.3%) ≥15 yr: *n* = 76 (16.0%)	ELISA parvovirus B19 IgG Biotrin International Ltd., Dublin, Ireland
^†^ N.A. = not available ^‡^ ELISA = enzyme-linked immunosorbent assay							

**Table 2:** The summary of the parvovirus B19 seroprevalence and risk estimates for daycare workers.

	Risk Estimates (Outcomes Ia and Ib)				Seroprevalence Estimates (Outcome II)		
Study ID	Effect Estimate	Effect Value	Adjusted for	Further Analysis	Age Category	Seroprevalence, % in Daycare Workers	Other Analysis
De Villemeur et al. 2011	PR ^†^ (Outcome Ib)	1.05 (0.94–1.16)	Age, occupational group, number of own children, attendance/duration of in-home childcare and/or informal child-care and/or child-care facility, and residence in a country of low/medium economic development	-	all	79.4 (75.1–83.3)	-
					≤37 years	67.7 (59.0–75.5)	
					>37 years	85.4 (80.6–89.5)	
Riipinenet al. 2014	HR ^‡^ (Outcome Ia)	Daycare worker HR = 2.63 (1.27–5.46) Other occupation HR = 0.85 (0.18–4.09) Reference: health care workers	Age, employment, number of children, high infection risk period, capital region	Nulliparous women only: HR = 5.59 (1.40–22.4)	all	59.1 (56.9–61.3)	-
					19–34 years	58.9 (56.6–61.2)	
					35–48 years	61.3 (53.8–68.5)	
Van Rijckevorsel et al. 2012	PR ^†^ (Outcome Ib)	1.2 (1.1–1.4)	Age, country of birth, having children	-	all	72.2 (66.7–78.2)	-
Gilbert et al. 2005	-	-	-	-	all	69.8 (65.5–73.9)	Seroprevalence by experience in daycare: <5 yr: 61.1% (95% CI 53.1–68.7) 5–9 yr: 71.2% (95% CI 62.7–78.8) 10–14 yr: 75.5% (95% CI 66.2–83.3) ≥15 yr: 77.6% (95% CI 66.6–86.4)
					≤34 years	64.6 (58.2–70.6)	
					>34 years	75.5 (69.5–81.0)	
^†^ Prevalence Ratio ^‡^ Hazard Ratio							

## Supplementary Materials

The following are available online at https://www.mdpi.com/1660-4601/16/8/1392/s1, Table S1: Risk of bias form; Figure S1: Funnel plot of studies in meta-analysis; Figure S2: Parvovirus B19 seroprevalence (%) of all included studies; Figure S3: Parvovirus B19 seroprevalence (%) by age.
