# Supplementary material for: Are Daycare Workers at a Higher Risk of Parvovirus B19 Infection? A Systematic Review and Meta-Analysis
Source: Int J Environ Res Public Health. 2019 Apr 17;16(8):1392. doi: 10.3390/ijerph16081392 (PMC6517978; doi:10.3390/ijerph16081392)
Supplement: Supplementary file 1 [file ijerph-16-01392-s001.pdf]

SUPPLEMENTARY MATERIAL

Risk of Bias Assessment

(i) Recruitment procedure and follow-up. In order to be rated as low risk, the recruitment of all study types must have evaded selection bias. For cross-sectional studies and cohort studies, if the daycare worker and daycare center response was acceptable (50% or more), or if the response was between 30% and 50% and a non-responder analysis was done to exclude substantial differential selection, the risk of bias was rated as low for this domain. For cohort studies, if the loss to follow-up was below 20%, and there was no substantial difference between the comparison groups the risk of bias was rated as low for this domain. Case-control studies had the same response requirements for cases and controls.

(ii) Exposure definition and measurement. If the exposure definition included at least basic job characteristics (i.e. job tasks or length of employment), if the exposure was accurately measured to minimize bias, and if an adequate comparison group of non-exposed workers (i.e. office workers) was used, this domain was considered to be at low risk of bias. If different methods were used to measure exposure in different groups (or in case and control subjects), this domain was considered to have a high risk of bias. For the rate or risk outcomes, if only an inadequate comparison group was used which would not reflect the general population (i.e. healthcare workers), this domain was rated as having a high risk of bias.

(iii) Outcome source and validation. If the outcome was objectively measured (i.e. by positive serology per parvovirus B19 IgG ELISA, used according to test kit instructions) and if measurement methods were similar in the different population groups, this domain was rated as having low risk of bias.

(iv) Confounding and effect modification. If major confounding factors (at least age and socioeconomic status) were considered when calculating risk estimators, the study was considered to have a low risk of bias. Adjusting for age and socioeconomic status is important because of their effect on parvovirus B19 seroprevalence (4, 9). Gender was not considered as no difference on parvovirus B19 infection has been seen between men and women (20).

(v) Analysis methods. If authors used adequate statistical models to reduce bias (i.e. standardization, matching, adjustment in a multivariate model, or stratification), this domain was declared to have low risk of bias. For studies reporting parvovirus B19 seroprevalence or incidence, the sex and age characteristics of the population must be described.

(vi) Chronology. For the parvovirus B19 risk and rate outcomes, if the negative serology was objectively measured at baseline, this domain was considered to have a low risk of bias. For parvovirus B19 seroprevalence, the chronology domain was not evaluated as cross-sectional studies were considered appropriate.

(vii) Funding. This was assessed in two areas: sources of funding and the involvement of the funding body in the research. If a study was funded by non-profit organization(s) and the study was not affected by sponsors, the domain was rated as low risk of bias. If the sponsoring organization participated in the data analysis or the study was probably affected by the sponsors, this domain was considered as having a high risk of bias.

(viii) Conflict of interest. If the authors reported not having conflict of interest or if it was clear from either the report or communication that the study was not affected by the authors' affiliation, this domain was rated as having low risk of bias. If at least one author had a conflict of interest, this domain was considered as having a high risk of bias.

Table S1. Risk of bias form.

Major risk of bias domains*	Risk	Criteria
**1. Recruitment procedure & follow-up (in cohort studies)**:<br><br>***For cohort studies***<br>*HINT: We are looking for selection bias:*<br>- *Was the cohort representative of a defined population? #*<br>- *Was everybody included who should have been included? #*<br>- *If response rate on day care centre level is slightly <50% but does not indicate selection bias, it will be listed as a demerit in extraction table.*<br><br>*PRELIMINARY RULING:*<br>- *If the cohort recruitment is based on a convenient/ self-reported sampling OR if response is <10%, the study will be excluded from analysis.*	low	☐ Cohort recruitment was acceptable.#<br>☐ Baseline response on both daycare workers and day care centre level is acceptable (50% or more) OR is <50% and >30%, but substantial differential selection could be excluded (e. g. by a non-responder analysis).<br>☐ Loss to follow-up is below 20% in total and not different between the two groups (up to 10% difference).*
	high	☐ Cohort recruitment was not acceptable.#<br>☐ Response not reported/ not calculable.<br>☐ Total loss to follow-up is larger than acceptable (20% or more)* OR drop out differs between the groups by more than 10%* OR the reasons for drop out considerably differ between exposed and non-exposed groups.*
For case-control studies<br>*HINT: We are looking for selection bias:*<br>- *Were the cases and control subjects representative of the same defined population ("study base"; geographically and/or temporally)? #*<br>- *Was there an established reliable system for selecting all the cases? #*<br>- *The same exclusion criteria are used for both cases and controls.*	low	☐ Case selection and recruitment was acceptable.#<br>☐ Control subjects' selection and recruitment was acceptable.#<br>☐ Baseline response for cases and control subjects is acceptable (50% or more) OR it is <50% and >30%, but substantial differential selection of cases and control subjects could be excluded (e.g. by a non-responder analysis)*
	high	☐ Case selection and recruitment was not acceptable.#<br>☐ Control subjects' selection and recruitment was not acceptable.#<br>☐ Non-response was >70% for cases or control subjects OR it was >50% and<70%, but substantial differential selection of cases and control subjects could not be excluded.*<br>☐ Response not reported/ not calculable

Major risk of bias domains*	Risk	Criteria
#<br>- *Comparison is made between participants and non-participants to establish their similarities or differences. #*<br>- *If response rate on day care centre level is slightly <50% but does not indicate selection bias, it will be listed as a demerit in extraction table.*<br><br>*PRELIMINARY RULING:*<br>- *If the cohort recruitment is based on a convenient/ self-reported sampling OR if response is <10%, the study will be excluded from analysis.*		
For cross-sectional studies<br><br>*HINT: We are looking for selection bias:*<br>- *Was the study population representative of a defined population? #*<br>- *Was everybody included who should have been included? #*<br>- *If response rate on day care centre level is slightly <50% but does not indicate selection bias, it will be listed as a demerit in extraction table.*<br><br>*PRELIMINARY RULING:*<br>- *If the cohort recruitment is based on a convenient/ self-reported sampling OR if response is <10%, the*	low	☐ Recruitment of the study population was acceptable.#<br>☐ Non-response was less than 50% OR it was >50% and <70%, but substantial differential selection of the study population could be excluded (e.g. by a non-responder analysis).*
	high	☐ Recruitment of the study population was not acceptable.#<br>☐ Non-response was >70% OR it was >50% and <70%, but substantial differential selection of the study population could not be excluded.*<br>☐ Response not reported/ not calculable.

Major risk of bias domains*	Risk	Criteria
study will be excluded from analysis.		
**2. Exposure definition and measurement**	low	☐ Exposure definition included at least basic job characteristics (e.g., job tasks, length of employment).<br>☐ Exposure was accurately measured to minimize bias.[#]<br>☐ Adequate comparison group of non-exposed workers (e.g. office workers) included.
	high	☐ Exposure does not cover basic job characteristics.<br>☐ Exposure was not accurately measured.[#]<br>☐ Different methods were used to measure exposure in different groups/ cases and control subjects (***in case-control studies***).[§]<br>☐ No adequate comparison group of non-exposed workers included ***(only for outcome 1b)***
	unclear	☐ Not reported.
**3.Ia Outcome "seroconversion rate". Source and validation**	low	☐ Outcome was accurately/ objectively measured to minimize bias (positive serology, medical diagnosis).[#]<br>☐ Measurement methods were similar in the different groups.[#]
	high	☐ Outcome was not accurately or subjectively measured (self-reported).[#]<br>☐ Measurement methods were different in the groups.[#]
	unclear	☐ Not reported.
**3.Ib Outcome "prevalence ratio or prevalence odds ratio ". Source and validation**	low	☐ Outcome was accurately/ objectively measured to minimize bias (e.g. positive serology, medical diagnosis).[#]<br>☐ Measurement methods were similar in the different groups.[#]
	high	☐ Outcome was not accurately or subjectively measured (e.g. self-reported).[#]<br>☐ Measurement methods were different in the groups.[#]
	unclear	☐ Not reported.
**3.II Outcome "seroprevalence of the daycare workers". Source and validation.**	low	☐ Outcome was accurately/ objectively measured to minimize bias (e.g. positive serology).[#]
	high	☐ Outcome was not accurately or subjectively measured.[#]
	unclear	☐ Not reported.
**4. Confounding and effect modification** *HINT: If the immunity status of the children in care is not being considered, it will be listed as a demerit in extraction table.*	low	☐ If risk estimators were calculated, major confounding factors (at least age and SES) were considered.<br>☐ If only prevalence or incidence was assessed, at least age is described.
	high	☐ Major confounding factors or effect modifiers were not considered.
	unclear	☐ Not reported.
**5. Analysis method:**	low	☐ Authors used adequate statistical models to reduce bias (e.g.,

Major risk of bias domains*	Risk	Criteria
**methods to reduce research specific bias**<br>*HINT: If the prevalence of serology is very high, we will not accept Prevalence Odds Ratios as adequate.*		standardization, matching, adjustment in multivariate model, stratification, propensity scoring).§ For prevalences, matching/stratification may not be required as long as a good description of the age structure and immunization status of the population is given.
	high	☐ Authors did not use adequate statistical models to reduce bias.
	unclear	☐ Not reported

Minor risk of bias domains*	Risk	Criteria
**6. Chronology**	low	☐ Incident diseases were included.#<br>☐ Temporal relation may be established (exposure precedes the outcome).#<br>☐ Negative serology known at baseline (career entry, baseline of study) AND was accurately/ objectively measured.<br>☐ For outcomes 2 and 3, cross-sectional studies are appropriate.
	high	☐ Prevalent diseases were included OR prevalent diseases of baseline were not excluded ***(in cohort studies)***.#<br>☐ Temporal relation cannot be established.<br>☐ Serology is unknown at baseline.<br>☐ Cross-sectional studies without basic information about temporal course (not applicable to outcomes 2 or 3)
	unclear	☐ Not reported.
**7. Funding**	low	☐ Grant/ non-profit-organizations*<br>☐ Study was clearly not affected by sponsors.*
	high	☐ Sponsoring organization participated in data analysis.<br>☐ Study was probably affected by sponsors.
	unclear	☐ Industry, combined industry+grant*, unclear if study was affected by sponsors.<br>☐ Not reported.
**8. Conflict of interest**	low	☐ Reported not having conflict of interest or clear from report/ communication that study was not affected by author(s) affiliation.*
	high	☐ Conflict of interest exists (at least one author).*
	unclear	☐ Not reported.

**Overall risk of bias assessment:**		**Low Risk**	**High Risk**	**Unclear Risk**
Major domains	1. Recruitment procedure & follow-up (in cohort studies)			
	2. Exposure definition and measurement			

	3.Ia Outcome "seroconversion rate". Source and validation			
	3.Ib Outcome "prevalence ratios or prevalence odds ratios ". Source and validation			
	3.II Outcome "seroprevalence of the daycare workers". Source and validation			
	4. Confounding and effect modification			
	5. Analysis method: methods to reduce research specific bias			
Minor domains	6. Chronology			
	7. Funding			
	8. Conflict of interest			
**General rule for rating:**	**Low risk of bias:** low risk in all major domains **High risk of bias:** if not low risk — **Overall assessment:**			
*according to Ijaz et al. (2013), with modifications<br># SIGN/CASP<br>§ Shamiliyan et al (2011), with modifications				

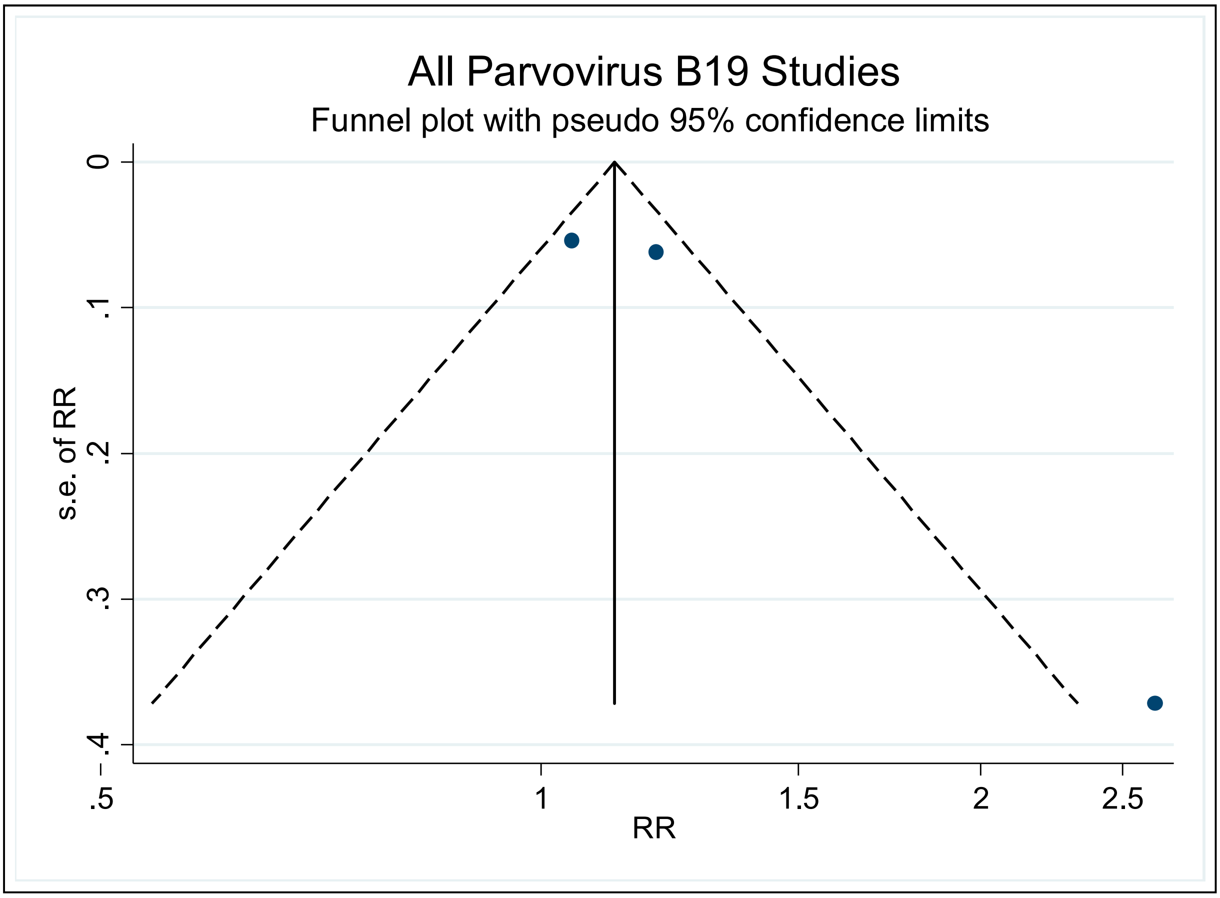

Figure S1. Funnel plot of studies in meta-analysis

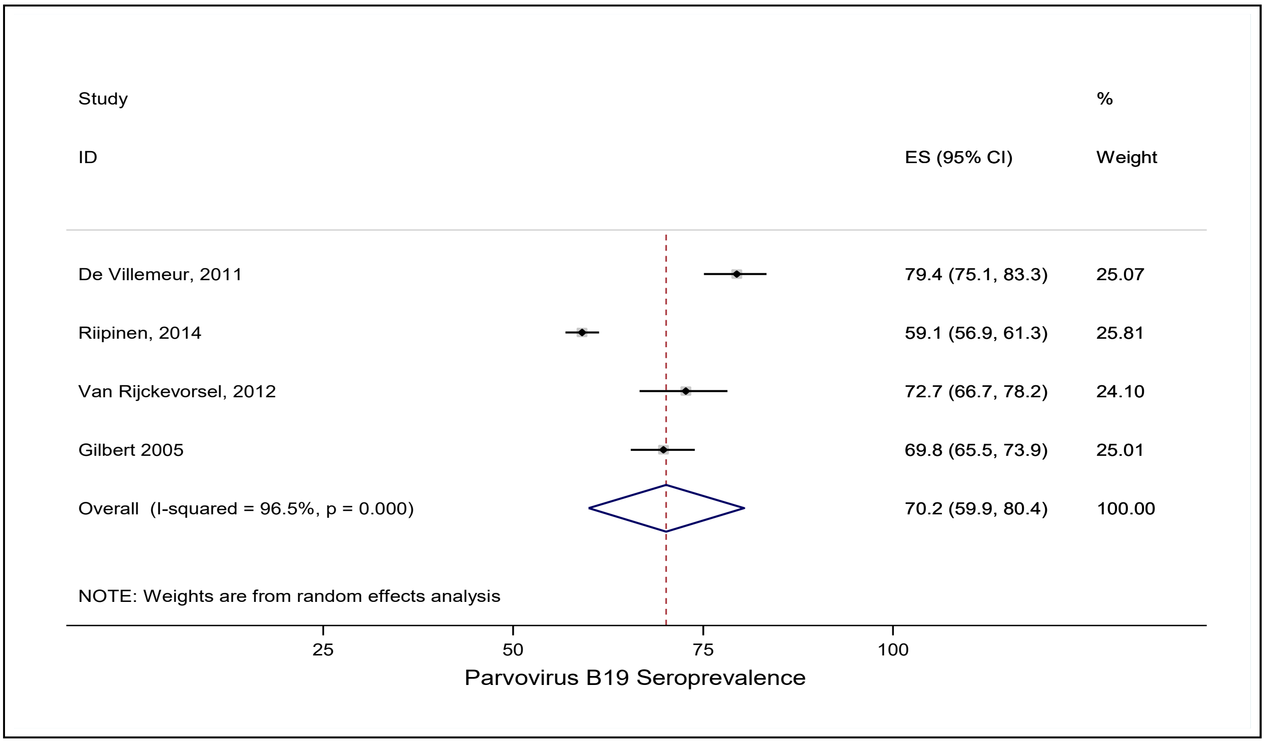

Figure S2. Parvovirus B19 seroprevalence (%) of all included studies.

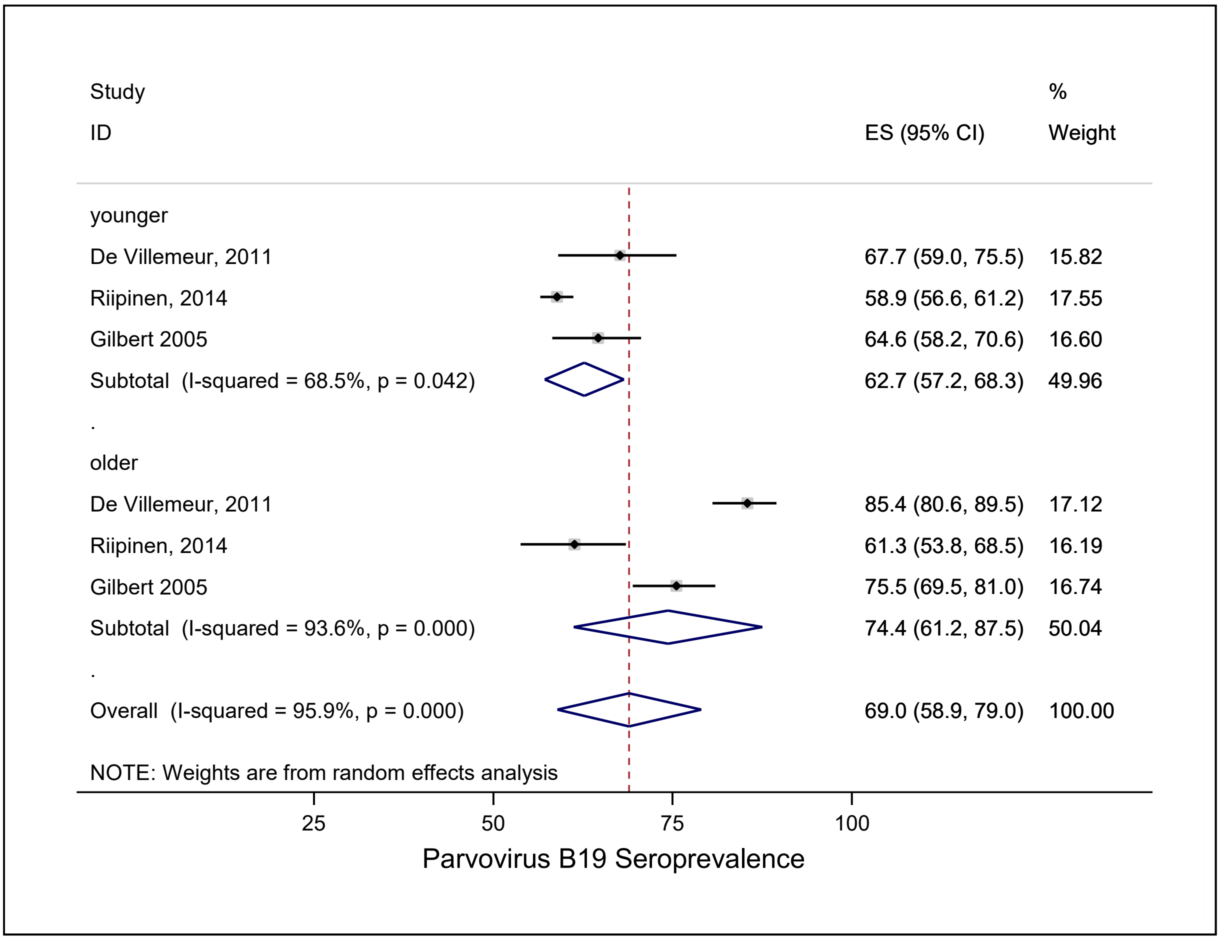

Figure S3. Parvovirus B19 seroprevalence (%) by age.
